# The New Journal of Dentistry

**Published:** 1884-11

**Authors:** 


### The New England Journal of Dentistry.

This journal contains, on its editorial page, the following: "We have to announce that this number will close the existence of the new *Journal of Dentistry, per se.* Arrangements are perfected by which this journal and the *Archives of Dentistry* are to consolidate.

Several months since, the Dental Journal Association was organized and chartered by leading members of the profession in the West, for the purpose of publishing an independent dental journal. It purchased, as a nucleus, the *Missouri Dental Journal,* and has issued its ninth number under the title of the *Archives of Dentistry.* After mature deliberation, the publishers and editors of the new *Journal of Dentistry* have decided that the interest of the profession may be best served by uniting their forces with this association, and for the purpose of giving our subscribers an opportunity to form the acquaintance of the combined journal, they will be supplied with it for the balance of the year. The Association will have the entire control of the editorial work of the journal, and arrangements have been made with

J. H. Chambers & Co., of St. Louis, Chicago, and Atlanta, for a term of years, for its publication. The whole country is being organized for the editorial work, and no effort will be spared of editors or publishers, to make the *Archives* a national journal, acceptable in every sense to the profession."

This movement will certainly strike the profession, for the most part at least, with surprise, as it was supposed that the *New England Journal* had been established as a permanent institution. New England ought certainly to have a dental journal within its territory. We do not suppose that this enterprise failed for want of support, but the change is perhaps made for prudential reasons, or because it was thought more good could be accomplished by combining these journals, and having the members of its editorial corps distributed throughout the country, so that every thing of value may be gathered and presented to the profession through this medium.

If this enterprise is to be carried out, as we may properly infer from this proposed arrangement, what will become of the other journals? What purpose can they serve, except to retail at second hand, material from this national independent journal?

We trust, however, that there will not be a disposition on the part of the management of the *Archives of Dentistry* to absorb every thing, and that at least a little gleaning will be left for others less pretentious; but seriously, we look for great things from the *Archives of Dentistry*. When the East, the West, and the South combine, something out of the ordinary course may be expected.

